# Supplementary material for: Feed efficiency and maternal productivity of Bos indicus beef cows
Source: PLoS One. 2020 Jun 3;15(6):e0233926. doi: 10.1371/journal.pone.0233926 (PMC7269248; doi:10.1371/journal.pone.0233926)
Supplement: S1 Table — (DOCX) [file pone.0233926.s001.docx]

**S1 Table. Descriptive statistics for performance traits, milk yield and blood metabolites of Nellore cows evaluated from 22±5 to 102±7 days of lactation**

| Trait | Mean (±SD) | Min | Max |
| --- | --- | --- | --- |
| Initial weight, kg | 484±41 | 394.80 | 570.19 |
| RFI, kg/day | 0±1.01 | -3.19 | 3.40 |
| DMI cow, kg/day | 12.4±1.5 | 8.96 | 16.1 |
| DMI calf, kg/day | 1.61±0.405 | 0.818 | 2.31 |
| BW^0.75^, kg | 107±6 | 92.3 | 118 |
| ADG, kg/day | 0.6317±0.32 | 0.0198 | 1.332 |
| MY_63_, kg | 8.77±2.6 | 4.50 | 19.64 |
| ECMY_63_, kg | 11.61±4.1 | 4.22 | 30.51 |
| Milk fat_63_, % | 5.43±1.4 | 1.64 | 10.4 |
| Milk protein_63_, % | 3.9±0.3 | 3.27 | 4.43 |
| Milk lactose_63_, % | 4.96±0.2 | 4.63 | 5.38 |
| MY_82_, kg | 7.38±1.6 | 1.58 | 10.40 |
| ECMY_82_, kg | 10.0±2.3 | 1.59 | 14.49 |
| Milk fat_82_, % | 5.75±1.4 | 3.44 | 9.89 |
| Milk protein_82_, % | 4.00±0.3 | 3.16 | 4.62 |
| Milk lactose_82_, % | 4.89±0.3 | 3.76 | 5.54 |
| 12th-13th rib fat thickness, mm | 6.02±2.2 | 2.30 | 9.80 |
| Longitudinal 11th-13th rib fat thickness, mm | 5.61±1.6 | 2.65 | 9.40 |
| Transverse plane of the flank fat thickness, mm | 6.12±1.5 | 2.45 | 10.33 |
| Median transverse plane hook bone to pin bone fat thickness, mm | 8.56±2.5 | 3.07 | 16.10 |
| Rump fat thickness, mm | 7.81±2.6 | 2.42 | 14.85 |
| Glucose_15_, mg/dL | 95.7±18 | 69.7 | 170 |
| Cholesterol_15_, mg/dL | 152±38 | 85.3 | 242 |
| Triglycerides_15_, mg/dL | 37.2±15 | 10.0 | 63.7 |
| β-hydroxybutyrate_15_, mmol/L | 0.59±0.4 | 0.12 | 2.65 |
| Albumin_15_, g/dL | 4.04±0.8 | 2.85 | 6.01 |
| Urea_15_, mg/dL | 51.6±22 | 17.7 | 119 |
| Creatinine_15_, mg/dL | 1.31±0.8 | 0.23 | 2.52 |
| Calcium_15_, mg/dL | 10.1±1.9 | 7.75 | 14.7 |
| Phosphorus_15_, mg/dL | 6.27±1.8 | 2.74 | 9.62 |
| Magnesium_15_, mg/dL | 2.92±0.5 | 1.99 | 4.25 |
| Cortisol_15_, ug/dL | 35.1±21 | 3.54 | 105 |
| Insulin_15_, μUI/mL | 0.73±0.9 | 0.22 | 5.14 |
| Glucose_41_, mg/dL | 91.3±16 | 68.6 | 138 |
| Cholesterol_41_, mg/dL | 189±55 | 108 | 307 |
| Triglycerides_41_, mg/dL | 36.7±13 | 16.2 | 65.1 |
| β-hydroxybutyrate_41_, mmol/L | 0.75±0.4 | 0.10 | 1.85 |
| Albumin_41_, g/dL | 4.60±1.2 | 2.26 | 7.36 |
| Urea_41_, mg/dL | 62.7±27 | 18.4 | 144 |
| Creatinine_41_, mg/dL | 1.36±0.6 | 0.32 | 2.41 |
| Calcium_41_, mg/dL | 9.18±2.5 | 4.79 | 17.1 |
| Phosphorus_41_, mg/dL | 6.10±1.6 | 2.92 | 9.37 |
| Magnesium_41_, mg/dL | 2.80±0.4 | 1.97 | 4.23 |
| Cortisol_41_, ug/dL | 28.7±15 | 6.96 | 68.3 |
| Insulin_41_, μUI/mL | 0.55±0.2 | 0.19 | 1.54 |
| Glucose_62_, mg/dL | 94.6±16 | 56.6 | 152 |
| Cholesterol_62_, mg/dL | 227±63 | 116 | 372 |
| Triglycerides_62_, mg/dL | 29.1±7.2 | 15.8 | 52.6 |
| β-hydroxybutyrate_62_, mmol/L | 0.76±0.4 | 0.14 | 1.65 |
| Albumin_62_, g/dL | 5.08±1.0 | 3.03 | 6.74 |
| Urea_62_, mg/dL | 73.3±26 | 30.2 | 140 |
| Creatinine_62_, mg/dL | 1.69±0.4 | 0.91 | 2.56 |
| Calcium_62_, mg/dL | 7.83±1.7 | 2.36 | 13.1 |
| Phosphorus_62_, mg/dL | 5.94±1.0 | 4.14 | 8.15 |
| Magnesium_62_, mg/dL | 2.91±0.6 | 1.88 | 4.16 |
| Cortisol_62_, ug/dL | 27.8±16 | 5.34 | 70.4 |
| Insulin_62_, μUI/mL | 0.72±0.6 | 0.24 | 3.92 |

_N_Subscript number after the name of traits means the day (day of lactation) of measurement.
