# Supplementary material for: Feed efficiency and maternal productivity of Bos indicus beef cows
Source: PLoS One. 2020 Jun 3;15(6):e0233926. doi: 10.1371/journal.pone.0233926 (PMC7269248; doi:10.1371/journal.pone.0233926)
Supplement: S2 Table — (DOCX) [file pone.0233926.s002.docx]

**S2 Table. Descriptive statistics for milk yield and blood metabolites of Nellore cows evaluated from 103±7 to 190±13 days of lactation**

| Trait | Mean (±SD) | Min | Max |
| --- | --- | --- | --- |
| 12th-13th rib fat thickness, mm | 8.32±3.1 | 2.00 | 13.7 |
| Longitudinal 11th-13th rib fat thickness, mm | 8.96±2.8 | 2.35 | 15.6 |
| Transverse plane of the flank fat thickness, mm | 9.76±2.3 | 2.98 | 13.5 |
| Median transverse plane hook bone to pin bone fat thickness, mm | 14.0±3.9 | 5.45 | 23.4 |
| Rump fat thickness, mm | 13.2±4.7 | 3.72 | 24.7 |
| MY_152_, kg | 6.63±1.7 | 2.43 | 9.18 |
| ECMY_152_, kg | 9.76±2.7 | 3.85 | 14.2 |
| Fat milk_152_, % | 6.41±1.3 | 4.33 | 11.3 |
| Protein milk_152_, % | 4.42±0.4 | 2.40 | 5.20 |
| Lactose milk_152_, % | 4.71±0.2 | 4.37 | 5.10 |
| Glucose_120_, mg/dL | 87.55±12 | 67.2 | 121 |
| Cholesterol_120_, mg/dL | 221±54 | 133 | 405 |
| Triglycerides_120_, mg/dL | 28.4±11 | 15.0 | 70.4 |
| β-hydroxybutyrate_120_, mmol/L | 0.66±0.3 | 0.10 | 1.40 |
| Albumin_120_, g/dL | 5.21±1.2 | 2.97 | 9.29 |
| Urea_120_, mg/dL | 102±27 | 55.8 | 179 |
| Creatinine_120_, mg/dL | 1.80±0.2 | 1.22 | 2.38 |
| Calcium_120_, mg/dL | 9.49±4.9 | 3.93 | 18.6 |
| Phosphorus_120_, mg/dL | 6.42±1.2 | 4.54 | 10.0 |
| Magnesium_120_, mg/dL | 2.86±1.0 | 1.91 | 8.41 |
| Cortisol_120_, ug/dL | 19.9±15 | 5.13 | 59.2 |
| Insulin_120_, μUI/mL | 0.89±0.8 | 0.28 | 4.63 |

_N_Subscript number after the name of traits means the day (day of lactation) of measurement.
