# Supplementary material for: Feed efficiency and maternal productivity of Bos indicus beef cows
Source: PLoS One. 2020 Jun 3;15(6):e0233926. doi: 10.1371/journal.pone.0233926 (PMC7269248; doi:10.1371/journal.pone.0233926)
Supplement: S3 Table — (DOCX) [file pone.0233926.s003.docx]

**S3 Table. Spearman correlation coefficients of milk yield and blood plasma metabolites of cows between 22±5 to 102±7 days of lactation and 22±5 to 190±13 days of lactation**

| Trait | Correlation (P-value) |
| --- | --- |
| ECMY | 0.70 (<0.0001) |
| Glucose | 0.89 (<0.0001) |
| Cholesterol | 0.92 (<0.0001) |
| Triglycerides | 0.69 (<0.0001) |
| β-Hydroxybutyrate | 0.92 (<0.0001) |
| Albumin | 0.88 (<0.0001) |
| Urea | 0.84 (<0.0001) |
| Creatinine | 0.95 (<0.0001) |
| Calcium | 0.30 (0.0315) |
| Phosphorus | 0.89 (<0.0001) |
| Magnesium | 0.24 (0.0778) |
| Cortisol | 0.88 (<0.0001) |
| Insulin | 0.85 (<0.0001) |

ECMY: energy-corrected milk yield.
