# Supplementary material for: Feed efficiency and maternal productivity of Bos indicus beef cows
Source: PLoS One. 2020 Jun 3;15(6):e0233926. doi: 10.1371/journal.pone.0233926 (PMC7269248; doi:10.1371/journal.pone.0233926)
Supplement: S4 Table — (DOCX) [file pone.0233926.s004.docx]

**S4 Table. Pearson correlation among the components of feed efficiency with average of milk yield and blood metabolites evaluated from 22±5 to 102±7 days of lactation**

| Trait | DMI_22- 102_ | ADG_22- 102_ | BW^0.75^_22- 102_ | RFI_22- 102_ |
| --- | --- | --- | --- | --- |
| MY_22- 102_ | 0.00 | 0.29* | 0.009 | -0.09 |
| ECMY_22- 102_ | 0.03 | 0.15 | 0.10 | -0.09 |
| Glucose_22- 102_ | -0.14 | -0.10 | -0.09 | -0.02 |
| Cholesterol_22- 102_ | 0.18 | -0.43* | 0.36* | -0.03 |
| Triglycerides_22- 102_ | 0.005 | 0.49* | -0.25 | 0.06 |
| β-Hydroxybutyrate_22- 102_ | -0.04 | 0.55* | -0.09 | -0.04 |
| Albumin_22- 102_ | 0.17 | -0.51* | 0.43* | -0.07 |
| Urea_22- 102_ | 0.31* | -0.28* | 0.44* | 0.12 |
| Creatinine_22- 102_ | 0.07 | -0.58* | 0.29* | -0.05 |
| Calcium_22- 102_ | -0.03 | 0.16 | -0.12 | -0.12 |
| Phosphorus_22- 102_ | 0.10 | -0.54* | 0.30* | 0.01 |
| Magnesium_22- 102_ | -0.15 | 0.06 | -0.22 | -0.05 |
| Cortisol_22- 102_ | 0.05 | -0.28* | 0.01 | 0.20 |
| Insulin_22- 102_ | 0.04 | -0.13 | 0.13 | -0.03 |

*P<0.05.
