# Supplementary material for: Feed efficiency and maternal productivity of Bos indicus beef cows
Source: PLoS One. 2020 Jun 3;15(6):e0233926. doi: 10.1371/journal.pone.0233926 (PMC7269248; doi:10.1371/journal.pone.0233926)
Supplement: S5 Table — (DOCX) [file pone.0233926.s005.docx]

**S5 Table. Pearson correlation among the components of feed efficiency with milk yield and blood metabolites evaluated from 22±5 to 190±13 days of lactation**

| Trait | DMI_22- 190_ | ADG_22- 190_ | | BW^0.75^_22- 190_ | RFI_22- 190_ |
| --- | --- | --- | --- | --- | --- |
| MY_22-190_ | 0.09 | -0.18 | 0.13 | | 0.07 |
| ECMY_22- 190_ | 0.05 | -0.16 | 0.16 | | 0.08 |
| Glucose_22- 190_ | -0.02 | -0.09 | -0.06 | | -0.18 |
| Cholesterol_22- 190_ | 0.18 | -0.29* | 0.31* | | -0.12 |
| Triglycerides_22- 190_ | -0.19 | 0.18 | -0.09 | | -0.04 |
| β-Hydroxybutyrate_22- 190_ | -0.23 | 0.39* | -0.04 | | -0.14 |
| Albumin_22- 190_ | 0.24 | -0.37* | 0.27* | | -0.09 |
| Urea_22- 190_ | 0.38* | -0.13 | 0.26 | | 0.16 |
| Creatinine_22- 190_ | 0.18 | -0.51* | 0.12 | | 0.02 |
| Calcium_22- 190_ | -0.19 | 0.43* | -0.15 | | -0.03 |
| Phosphorus_22- 190_ | 0.32* | -0.34* | 0.15 | | 0.09 |
| Magnesium_22- 190_ | -0.04 | 0.12 | 0.04 | | -0.005 |
| Cortisol_22- 190_ | 0.19 | -0.30* | -0.02 | | 0.26 |
| Insulin_22- 190_ | 0.09 | -0.18 | 0.14 | | -0.002 |

*P<0.05.
