# Supplementary material for: Feed efficiency and maternal productivity of Bos indicus beef cows
Source: PLoS One. 2020 Jun 3;15(6):e0233926. doi: 10.1371/journal.pone.0233926 (PMC7269248; doi:10.1371/journal.pone.0233926)
Supplement: Attachment — Submitted filename: Response to Reviewer-R2.pdf [file pone.0233926.s007.pdf]

Dear Dr. Marcio de Souza Duarte
Academic Editor – PLoS ONE

We would like to thank you again, associate editor and #1 reviewer, for your time and effort to improve the quality of the manuscript.
We followed all recommendation of the Reviewer#1.
Changes made to the previous version are in the "Revised Manuscript with Track Changes-R2" file.
Below are the answers to the Reviewer #1.

Sincerely yours,

Maria Eugênia Mercadante, PhD
Instituto de Zootecnia

Reviewer #1

Reviewer #1: The manuscript has been improved. Thanks for the extensive revisions to improve the paper. I am OK with splitting the cattle into RFI groups but I do believe that it masks many of the effects and does not result int the data being used to its fullest extent. Yes it may be more practical to do this which may be more useful when presented to livestock producers but for scientific manuscripts analyzing data in a continuous fashion could result in a better explanation of the results (the shape of the curve of the relationship could be useful in understanding the physiology and for future prediction equations that could be developed). I will point out that just because there are many examples of publication breaking the data into RFI groups does not mean that it is the best way to analyze the data. I have a few other comments/suggestions for improvement. See below.

Line 75: This is an oversimplification of the concept of RFI. We have long known that there are many things besides BW that influence maintenance requirements. I would suggest rewording to be more careful with this interpretation of RFI.
AU: We changed the sentences to give suggestive idea rather than affirmative one.

Line 156: I think concept is supposed to be conceptus?
AU: Yes. It was changed.

Line 388: I would suggest saying after accounting for ADG and BW rather than saying that it is independent. That is a very strong word and in actuality the traits are not independent - it is how the data is analyzed.
AU: Ok, it was changed

Line 470: change saved to improved energy balance (or something like that)
AU: Ok, it was changed.

Line 519: This sentence is not clear. Please reword.
AU: The first sentence was changed to be clear.

Line 530: Although there is evidence of a positive relationship between ...
AU: Ok, it was changed.

Line 538: days of lactation
AU: Ok, it was changed.

Line 540: Despite the fact that cows in the presebt styudt were fed for ad libitum intake, negative RFI cows ...
AU: Ok, it was changed.

Line 557: This is highly dependent on the diet that the cows were fed. If I understand correctly, diets were formulated so that cows would be gaining maternal BW and BCS during the experiment. Because of this it may not be surprising that cows were not mobilizing body reserves. If a lower energy diet was fed, you likely would observe different results. This should be considered in this discussion.
AU: We added a commentary about the diet.

Line 569: use alternative wording than balancing act
AU: Ok, it was changed by “delicate balance”.

Line 583: need the last name of the author for the reference before 34.
AU: It was already ok in the final R1 version.
